# *Plasmodium falciparum* parasites lacking histidine-rich protein 2 and 3: a review and recommendations for accurate reporting

**DOI:** 10.1186/1475-2875-13-283

**Published:** 2014-07-22

**Authors:** Qin Cheng, Michelle L Gatton, John Barnwell, Peter Chiodini, James McCarthy, David Bell, Jane Cunningham

**Affiliations:** 1 Drug Resistance and Diagnostics, Australian Army Malaria Institute, Brisbane, Australia; 2 School of Public Health and Social Work, Queensland University of Technology, Brisbane, Australia; 3 Malaria Branch, Division of Parasitic Diseases and Malaria, Centers for Disease Control and Prevention, National Centre for Infectious Diseases, Atlanta, USA; 4 Hospital for Tropical Diseases, London School of Hygiene and Tropical Medicine, London, UK; 5 QIMR Berghofer Medical Research Institute, Brisbane, Australia; 6 Independent Consultant, Geneva, Switzerland; 7 Global Malaria Programme, World Health Organization, Geneva, Switzerland

## Abstract

Malaria rapid diagnostic tests (RDTs) play a critical role in malaria case management, surveillance and case investigations. Test performance is largely determined by design and quality characteristics, such as detection sensitivity, specificity, and thermal stability. However, parasite characteristics such as variable or absent expression of antigens targeted by RDTs can also affect RDT performance. *Plasmodium falciparum* parasites lacking the PfHRP2 protein, the most common target antigen for detection of *P. falciparum*, have been reported in some regions. Therefore, accurately mapping the presence and prevalence of *P. falciparum* parasites lacking *pfhrp2* would be an important step so that RDTs targeting alternative antigens, or microscopy, can be preferentially selected for use in such regions. Herein the available evidence and molecular basis for identifying malaria parasites lacking PfHRP2 is reviewed, and a set of recommended procedures to apply for future investigations for parasites lacking PfHRP2, is proposed.

## Role of malaria rapid diagnostic tests (RDTs)

Malaria remains endemic in 104 countries, representing a major public health problem in many [1]. While most countries are aggressively controlling malaria, and some are progressing towards elimination, much of the success relies on appropriate malaria case management based on early diagnosis and prompt treatment with efficacious anti-malarial drugs [2]. To improve case management the World Health Organization (WHO) recommends that parasitological confirmation by microscopy or malaria rapid diagnostic tests (RDTs) is conducted in all patients with a suspected diagnosis of malaria prior to commencing treatment [3].

The availability of quality-assured malaria RDTs in recent years has dramatically increased access to malaria diagnostics. Malaria RDTs are not only playing an increasing critical role in malaria case management, but also in malaria surveillance and case investigations in malaria elimination countries. They are also widely used in non-endemic settings to diagnose malaria in travellers returning from the tropics [4].

## Diagnostic targets of RDTs

Malaria RDTs are lateral flow devices that use antibodies to capture and detect parasite proteins by immunochromatography. Currently, over 200 brands of RDTs are commercially available for detecting different *Plasmodium spp.*[5]. Over the past five years 128 unique RDT products have been tested in the WHO-Foundation for Innovative New Diagnostics (FIND) Malaria RDT Evaluation Programme [6]. One hundred and ten (110) of the 127 RDTs that are capable of diagnosing *Plasmodium falciparum,* target an antigen that is unique to *P. falciparum*, the histidine-rich protein 2 (PfHRP2). Many antibodies used to detect PfHRP2 also detect histidine-rich protein 3 (PfHRP3), a protein that shares a high degree of similarity in amino acid sequence with PfHRP2 [7]. Indeed, the monoclonal antibodies in such tests target an epitope abundant in both proteins [7]. Generally, PfHRP2-detecting RDTs have better sensitivity than non-PfHRP2-detecting RDTs, [6] and tend to have greater thermal stability [8]. These considerations, combined with the higher number of available products meeting WHO procurement criteria [9] make PfHRP2-detecting RDTs a widely used diagnostic tool for *P. falciparum* infections.

## Use of RDTs

RDTs detecting *P. falciparum* alone are suitable for use in much of Western and sub-Saharan Africa where malaria cases are predominantly caused by *P. falciparum*. However, outside of Africa where *P. falciparum* co-exists with *Plasmodium vivax* and other Plasmodium species, RDTs that detect both *P. falciparum* and non-*falciparum spp*. are typically selected.

Forty-one of 44 African countries and 49 of 55 countries of other regions have adopted the WHO policy of parasitological diagnosis for all age groups in 2011 [1]. This was greatly facilitated by the roll out of quality assured RDTs. Globally, RDT sales reached a total of 205 million in 2012 (129 million PfHRP2, *P. falciparum*, only tests) [1].

## Performance of RDTs

The detection sensitivity of quality RDTs is generally similar to that of quality field microscopy. However, variable performance has been reported in field use [10]. False negative RDT results will delay anti-malarial treatment, potentially endangering life and the patient will be a source for ongoing malaria transmission.

## Possible causes for false negative test results

The major causes of false negative results can be grouped into five broad categories: poor product design or quality, unsuitable transport or storage conditions, parasite or operator factors and host parasite density/antigen concentration [11,12] (Table 1).

**Table 1:** Possible causes of false negative RDT results

**Classification**	**Cause of false negative RDT result**
Product design or quality	Poor detection sensitivity of a RDT product due to poor specificity, affinity, or insufficient quantity of antibodies used in the RDT
	Poor visibility of test bands due to high background colour on the test
	Incorrect instructions for use
Transport or storage conditions	Antibody degradation due to poor durability to heat or to incorrect transport or storage
Parasite factors	Variation in the amino acid sequence of the epitope targeted by the monoclonal antibody
	Parasites lacking the target antigens or expressing reduced levels of the target antigens
Operator factors	Operator error in preparing the RDT, performing the test, or interpreting the result
Host parasite density	Very low parasite densities/target antigen concentrations
	Prozone effect (hyperparasitemia/antigen overload)

Many of the potential causes can be eliminated or minimized by procuring good quality RDTs, by improving quality control of procured RDTs, and by good training of end users. However, host and parasite characteristics could be specific to the region where the RDTs are deployed and are beyond the realms of quality control and training. Therefore, false negative results should be exhaustively investigated, particularly in relation to parasites not expressing target antigens or expressing, as of yet unrecognized, antigenic variants. In regards to the potential contribution of the antigenic variants, Baker *et al.* found that despite extensive variations in the number and arrangement of various repeats encoded by the *pfhrp2* genes in parasite populations world-wide (458 isolates examined), no statistically robust correlation between gene structure and RDT detection rate for *P. falciparum* parasites at 200 parasites per microlitre was identified [13]. Thus, diversity of PfHRP2 does not appear to be a major cause of RDT sensitivity variation.

## Evidence review for parasites not expressing PfHRP2 and/or PfHRP3

When a parasite expresses little or no target antigen, a false negative result will occur. *P. falciparum* parasites not expressing PfHRP2 and/or PfHRP3 have been reported both in laboratory and field isolates. In contrast, there are no reports of parasites failing to express pLDH or aldolase, as these targets are essential enzymes for parasite metabolism and survival.

## Laboratory lines

*Plasmodium falciparum* parasites not expressing PfHRP2 were first reported in culture adapted, cloned laboratory lines: one originating from Papua New Guinea (FCQ27-D10) [14] and the other, from Thailand (Dd2) [15]. In both lines, the failure to express PfHRP2 resulted from a deletion of part [16] or entire protein encoding gene [15] due to breakage at chromosome ends (subtelomeric regions). Subpopulations of parasites deleting parts of the *pfhrp2* gene were also observed in a parasite line (Palo Alto) when cultured *in vitro*. These subpopulations showed different breaking points, which appear to be random within the exon 2 of *pfhrp2*[17].

PfHRP3 was reported missing in a parasite line originating from Honduras (HB3), again due to chromosomal breakage at the subtelomeric region, resulting in the deletion of *pfhrp3*[18]. Parasites with *pfhrp3* gene were favoured strongly in a genetic cross between 3D7 and HB3 compared to those without *pfhrp3*, due to rapid growth of progeny expressing PfHRP3 [18].

Parasites lacking both *pfhrp2* and *pfhrp3* genes were observed in a progeny of a genetic cross between Dd2 and HB3 [15]. This progeny inherited the Dd2 parental type at the *pfhrp2* locus on chromosome 8 and the HB3 parental type at *PfHRP3* locus on chromosome 13, thus causing a deletion of both genes. This parasite clone was able to grow and multiply *in vitro* with these deletions. Interestingly, presence of *pfhrp2* was not favoured in inheritance in the progeny [15].

## Field isolates

The first definitive report of *P. falciparum* parasites lacking PfHRP2 and/or PfHRP3 was published by Gamboa *et al.*[19]. This discovery was made as part of the WHO-FIND Malaria RDT Evaluation Programme in which over 300 field isolates were collected from different geographical areas and characterized for use in malaria RDT product testing at the Centers for Disease Control and Prevention (CDC), USA, and lot testing at the collecting laboratories. Amongst nine *P. falciparum* isolates collected in Iquitos, Peru, in 2007, eight were found to lack the *pfhrp2* and six to lack both the *pfhrp2* and *pfhrp3* genes when tested by PCR. When tested by ELISA, none of the eight parasites lacking *pfhrp2* had detectable PfHRP, but all had detectable pLDH [19]. Furthermore, parasites lacking both *pfhrp2* and *pfhrp3* returned negative results on three different brands of good quality RDTs.

**Figure 1:**
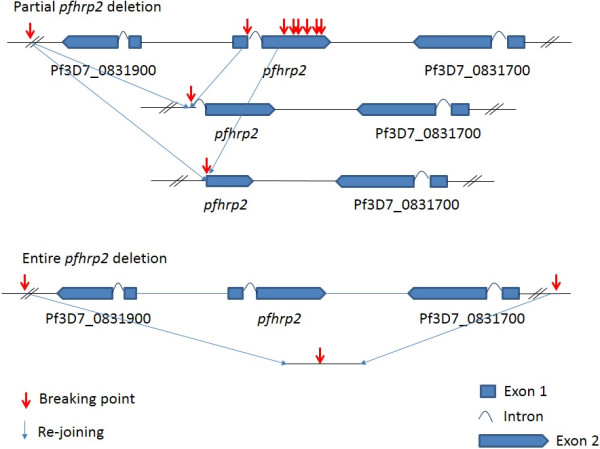
**Illustration of chromosome breakage points within and flanking *the pfhrp2 *** **gene and re-joining of the chromosome.**

DNA sequence analysis of the genes flanking *pfhrp2* and *pfhrp3* revealed that the lack of PfHRP2 was due to deletions on chromosome 8 which removed *pfhrp2* and upstream genes, while the lack of PfHRP3 was mostly due to deletions on chromosome 13 that removed *pfhrp3* and its flanking genes [19]. Deletions of both genes were characterized in more detail using a whole genome scanning approach in a different set of *P. falciparum* isolates also collected from the Iquitos region [20]. Genome scanning of 14 Peruvian *P. falciparum* isolates revealed deletions of subtelomeric regions in lengths of 20–25 kb containing several genes including *pfhrp2* and *pfhrp3*[20]. It should be noted that a truncation of *pfhrp3* exon 1 was also seen in two isolates causing non expression of PfHRP3 [19]. Although not reported to date in field isolates, a frame shift in these genes could also cause non-expression of the protein. Based on these published findings, deletions of *pfhrp2* occur because of chromosomal breakage and re-join in the subtelomeric regions of chromosome 8. The breaking point could occur in a broad region, often upstream of *pfhrp2* or within *pfhrp2* (Figure 1).

## Prevalence and distribution

After characterization and confirmation of *P. falciparum* parasites lacking PfHRP2 and PfHRP3 in Iquitos, Gamboa and colleagues [19] performed a retrospective investigation of the prevalence of these parasites in the Peruvian Amazon. 148 *P. falciparum* samples had been collected between 2003 and 2007 from various locations, and 41% and 70% of these samples lacked the *pfhrp2* and *pfhrp3* genes, respectively, with ≈ 22% of the parasites lacking both *pfhrp2* and *pfhrp3* genes [19].

Recently, Akinyi *et al.*[21] retrospectively investigated the genetic origins of *P. falciparum* lacking the *pfhrp2* gene in Peru. Surrounding Iquitos 13% of *P. falciparum* parasites collected between 1998 and 2001 had deleted *pfhrp2*. Prevalence increased to 40.6% in samples collected from the same area between 2003 and 2005 [19]. This is in perfect agreement with earlier findings [19]. Interestingly, Akinyi *et al.* showed that *pfhrp2* deletions occurred in 4 of the 5 genetic lineages in the 1998–2001 population, and in all 8 lineages in the 2003–2005 population [21]. This suggests that *pfhrp2* deletion has occurred multiple times in the Peruvian *P. falciparum* population and its prevalence has increased in recent years.

## A significant public health problem

*Plasmodium falciparum* parasites that lack part or all of the *pfhrp2* gene do not express the PfHRP2 protein and are, therefore, not detectable by PfHRP2-detecting RDTs. Although some of these parasites still have a functioning *pfhrp3* gene and produce PfHRP3, the lower abundance of PfHRP3 and the less sensitive detection of this protein with anti-PfHRP2 antibodies means that infection with such parasites may only be detectable by PfHRP2-detecting RDTs at higher parasitaemia (>1,000 parasites/μL) [22]. Therefore, a high prevalence of *P. falciparum* parasites lacking *pfhrp2* gene will cause a high rate of false negative results in PfHRP2-detecting RDTs. This is especially true in areas with low malaria transmission where multi-clone infections are rare, i.e. parasite lacking PfHRP2 are less likely co-infected with another strain having PfHRP2 in the same individual, and parasite densities are usually low. These false negative results will cause a major problem for malaria case management.

This effect is demonstrated in a recent study comparing sensitivities of several PfHRP2- and pLDH-based RDTs for detecting *P. falciparum* infections in health centres around Iquitos [23]. Samples used in this study included 19 (25.7% of the parasite isolates) parasite isolates lacking the *pfhrp2* gene. The sensitivity of 10 products based on PfHRP2 detection was approximately 70 – 72%, with none of the products detecting any of the 19 isolates containing parasites without *pfhrp2*. In contrast, four pLDH-detecting RDTs detected all 19 of these isolates, giving an average sensitivity of 97-99% [23]. Clearly, the sensitivity of PfHRP2-detecting RDTs was compromised in this region and they are not an appropriate diagnostic tool for diagnosing *P. falciparum* infections both there and in returning travellers from regions affected by HRP2 deletions. Instead, quality pLDH-detecting RDTs and microscopy should be used in this region of Peru, and in contiguous regions in bordering countries.

## Importance of correctly mapping parasites without *pfhrp2*

The potential impact of *P. falciparum* parasites lacking *pfhrp2* on malaria case management and on procurement decisions regarding the type of RDTs is significant, and as such it is imperative that the presence and prevalence of these parasites are accurately documented and mapped. However, incorrect or unsubstantiated reporting of parasites lacking *pfhrp2* may unnecessarily trigger a costly change of RDT product (although there is no guideline as to what prevalence level should trigger this change), a process which requires significant logistic and financial support, as well as user retraining. It may also damage overall user confidence in RDTs. So far, the rarity of reports summarized in Table 2 suggests *pfhrp2* deletion is not a major problem across broad areas; however, no systematic mapping has occurred. Not to be underestimated is the difficulty of determining the cause of false negative RDT results in the field, or when parasite antigen deletion may be suspected but there is poor capacity to investigate, and lack of guidance on how to conduct the investigation. The latter is addressed in the following sections.

**Table 2:** **Published studies reporting *P. falciparum *** **with deletions or no deletions of the *pfhrp2 *** **gene**

**Origin**			Source of samples*	**Initial evidence**			**Gene deletion analysis by PCR**			**Antigen analysis**		**Ref**	**Prevalence (no. of samples. year of collection)**
**Region**	**Country**	**Area**		**Microscopy**	**Quality RDT**	**Species PCR**	***pfhrp2 *** **(exon 1 & 2)**	**No. single copy genes**	**Flanking genes**	**HRP ELISA**	**2**^**nd **^**quality RDT**		
**South America**	Peru	Iquitos Condorcanqui Jaen San Lorenzo Yurimaguas	S	D	D	D	D	3	D	D	D	[19]	41% (148, 2003–2007)
				D	ND	D	D	3	ND	ND	ND		
		Iquitos surrounding	S	D	D	D	D	ND	ND	D	D	[23]	25.7% (74, 2010–2011)
		Iquitos Padre cocha Caballococha Bellavista	U	ND	ND	D	D	ND	D	ND	ND	[21]	20.7% (92, 1998–2001) 40.6% (96, 2003–2005)
	Brazil	Brazilian Amazon	S	D	D	D	D	ND	ND	ND	ND	[24]	Case report
	French Guiana		S	D	D	D	D	ND	ND	ND	ND	[25]	0% (140, 2009–2011)
**Africa**	Mali	Bamako	A/S	D	ND	D	D	1	ND	ND	ND	[26]	2% (480, 1996)
	DRC Gambia Kenya Mozambique Rwanda Tanzania Uganda		S	D	ND	D	Exon 2 only	ND	ND	D	ND	[27]	0% (77, 2–19 per country, 2005–2010)
	Senegal	Dakar	S	D	ND	D	D	1	ND	ND	ND	[28]	2.4% (136, 2009–2012)
**Asia**	India	Chhattisgarh	S	D	D	D	D	3	D	ND	D	[29]	4.2% (48, 2010)

*Source of samples: S = Symptomatic case, A = Asymptomatic case, U = not specified. D = done; ND = not done.

Note: Quality RDT indicates RDTs meet the WHO RDT recommended procurement criteria based on WHO Malaria RDT Product Testing.

## Evidence required to document *pfhrp2* deletion

Due to the potential consequences of *pfhrp2* deletion on malaria diagnosis at the clinical, public health and economic levels it is imperative that procedures and standards are developed in order to confirm new geographic foci of *pfhrp2-*deleted parasites. As a minimum, the following should be collected when reporting or documenting the presence of PfHRP2 deletions.

1. Initial evidence

• The sample is confirmed as microscopy positive for *P. falciparum,* the parasitaemia is counted and all findings are verified by two qualified microscopists and slide archived. The same sample does not produce a positive Pf band on two quality-assured PfHRP2-detecting RDTs taken from the same box of RDTs that produced positive results using a positive control, such as a known RDT positive blood sample. This sample should also be positive for the pan band if the RDT detects Pf and pan Plasmodium *spp*, and positive as Pf when a pLDH-detecting RDT meeting WHO procurement criteria is used. The requirement for a positive pan-band is because pan-bands are generally less sensitive than HRP2-test bands and therefore a positive pan-band, and negative HRP2 test band makes the possibility of a false negative due to low parasite density less probable.

• The sample is confirmed as *P. falciparum* by PCR and ideally quantified against the WHO International *P. falciparum* DNA standard [30] and the sample is negative for other *Plasmodium* species by PCR.

If all of the above initial evidence is observed, a confirmatory investigation should be undertaken. A blood sample (>50 μL) should be stored frozen or dried on filter papers (e.g. Whatman 3MM) or other collection cards as sources of DNA for confirmatory analyses outlined below. Blood slides and used RDTs could also be stored as sources of DNA.

2. Confirmatory evidence

• Gene deletion analysis

a. PCR to amplify the full length of exon 2 and the region across exon 1 and exon 2 of the gene (See Table 3 for suggested primer sequences), and fails to amplify a product in at least one experiment. This is designed to detect both entire and partial gene deletions based on known chromosome breaking points, See Figure 1 (many in exon 2).

**Table 3:** **Suggested primer sequences, PCR conditions and expected product sizes**[19,22]

**Gene name (previous ID)**	**Primer sequence**	**Annealing Tm**	**Expected product size**
Pf3D7_0831800	5′CAA AAG GAC TTA ATT TAA ATA AGA G3′(forward)	55°C	600 -1000 bp
*Pfhrp2* exon 2	5′ AAT AAA TTT AAT GGC GTA GGC A3′(reverse)		
	5′ ATT ATT ACA CGA AAC TCA AGC AC3′(nested forward)		
Pf3D7_0831800	5′ TAT CCG CTG CCG TTT TTG CC 3′	57°C	303 bp
*Pfhrp2* exon 1-2	5′ AGC ATG ATG GGC ATC ATC CTA 3′		
Pf3D7_0831700	5′ AGA CAA GCT ACC AAA GAT GCA GGT G 3′	60°C	227 bp
(MAL7P1_228)	5′ TAA ATG TGT ATC TCC TGA GGT AGC 3′		
Pf3D7_0831900	5′ TAT GAA CGC AAT TTA AGT GAG GCA G 3′	68°C	346 bp
(MAL7P1_230)	5′ TAT CCA ATC CTT CCT TTG CAA CAC C 3′		

b. PCR is able to amplify products for at least two single copy genes from the same sample. It does not matter on which chromosome these genes are located as the purpose is to verify the quality of DNA in the sample. While a PCR based on amplification of the 18sRNA gene is useful in confirming Plasmodium species, alone it does not provide sufficient evidence of DNA quality as it is a multi-copy gene, while *pfhrp2* is a single copy gene and, therefore, more susceptible to false negative test at low DNA concentration.

c. PCR targeting genes flanking *pfhrp2* amplifies none or only one of the flanking genes (Table 3 has suggested primers). Deletions of *pfhrp2* often include one or both of the genes flanking *pfhrp2*.

• Antigen analysis

a. To confirm that the parasite lacks the pfHRP2 protein, a second brand of quality PfHRP2-detecting RDT should be used to test the sample, and again a negative result should be produced. Ideally, this second RDT would use different antibodies, but this information is frequently not available.

b. Alternatively, a PfHRP2 ELISA could be performed, and again yield a negative result at a parasitaemia where the ELISA would be expected to be positive. A PfHRP2 variant with poor affinity for the antibodies used in the ELISA is conceivable but highly unlikely to occur and therefore, this step provides the most definitive proof of lack of functional protein due to partial or entire gene deletion. It should be noted that antigen analysis is also important for reporting no *pfhrp2* deletions because a frame shift within *pfhrp2* will also lead to a false negative RDT result, even if the gene is present.

3. Establish prevalence

Following confirmation of *pfhrp2* deletions in initial case investigations, surveillance of fever patients or community surveys around index cases should be carried out in the specific geographic region to determine the prevalence of parasites carrying gene deletions. Retrospective studies are an option if stored samples from the same region are available.

Outside Peru, prevalence of parasites lacking *pfhrp2* is almost certainly extremely low, but foci of deleted parasites may exist – the Peruvian deleted population went unrecognized until sample collection was undertaken for purposes other than case management. In such cases, a large number of samples may be required to establish reliable estimates of the prevalence of these parasites. In all cases, prevalence estimates should be accompanied by 95% confidence intervals. Procedures should be put in place for clinicians to collect appropriate samples when suspicious false negative cases arise, including frozen and dry blood spots samples,

## Summary of studies reporting *pfhrp2* deletions or no deletions

Since the first report of *P. falciparum* parasites lacking *pfhrp2* gene in 2010 [19], several publications have reported detection of these parasites in Peru and other areas [21,23,24,26,28,29]. The approaches used in these studies have varied. In Table 2, these studies are summarized against the recommended procedures outlined. The majority of studies meet most of the ‘initial evidence’ criteria, however, few follow up confirmatory tests are reported, including the quality of DNA, the deletion of flanking genes and antigen analysis.

## Conclusions

After reviewing published data we believe that unequivocal evidence exists for *pfhrp2* and *pfhrp3* gene deletions in *P. falciparum* in Peruvian Amazon at a high prevalence (40.6% in Iquitos and 2-40% in surrounding areas) [19,23]. Therefore, PfHRP2-detecting RDTs should not be used in this area of South America as a basis for treatment decisions. It is important that vigilance is high in other parts of Peru and of South America generally in order to determine the geographic spread of parasites with gene deletions.

In areas outside of South America, further studies should be undertaken to verify the existence, and if present, the prevalence and geographical extent of populations of parasites with gene deletions. Overwhelmingly, operational experience on RDT use indicates that there is no reason why PfHRP2-detecting RDTs should not be used as part of routine clinical case management in most malaria-endemic areas, they are safe and effective for case management [31-35]. Investigations should be carried out in cases where discrepant results are seen between different diagnostic methods that are not due to low parasite density in samples.

## Competing interests

The authors declare that they have no competing interests.

## Authors’ contributions

QC conducted the literature review and drafted the manuscript. MG carried out the data analysis and contributed to data interpretation. JB, JM, DB and PC contributed substantially to data interpretation and revising the draft manuscript. JC conceived of the study, participated in its design, data interpretation and in revising the draft manuscript. All authors read and approved the final manuscript.

## References

[B1] WHO World Malaria Report 2013 2013 Geneva: World Health Organization

[B2] WHO Antimalarial drug combination therapy. Report of a WHO technical consultation 2001 Geneva: World Health Organization

[B3] WHO Guidelines for the treatment of malaria 2010 2 Geneva: World Health Organization 25473692

[B4] Chilton D Malik AN Armstrong M Kettelhut M Parker-Williams J Chiodini PL Use of rapid diagnostic tests for diagnosis of malaria in the UK J Clin Pathol 2006 59 862 866 16603648 10.1136/jcp.2005.032904 PMC1860455

[B5] World Health Organization World Malaria Report 2012 2012 Geneva: World Health Organization

[B6] WHO Malaria rapid diagnostic test performance. Results of WHO product testing of malaria RDTs: Round 4 2012 Geneva: World Health Organization

[B7] Lee N Baker J Andrews KT Gatton ML Bell D Cheng Q McCarthy J Effect of sequence variation in *Plasmodium falciparum* histidine- rich protein 2 on binding of specific monoclonal antibodies: Implications for rapid diagnostic tests for malaria J Clin Microbiol 2006 44 2773 2778 16891491 10.1128/JCM.02557-05 PMC1594627

[B8] Chiodini PL Bowers K Jorgensen P Barnwell JW Grady KK Luchavez J Moody AH Cenizal A Bell D The heat stability of *Plasmodium* lactate dehydrogenase-based and histidine-rich protein 2-based malaria rapid diagnostic tests Trans R Soc Trop Med Hyg 2007 101 331 337 17212967 10.1016/j.trstmh.2006.09.007

[B9] WHO Information note on recommended selection criteria for procurement of malaria rapid diagnostic tests (RDTs) 2012 Geneva: World Health Organization http://www.who.int/malaria/publications/atoz/rdt_selection_criteria/en/

[B10] Abba K Deeks J Olliaro P Naing C Jackson S Takwoingi Y Donegan S Garner P Rapid diagnostic tests for diagnosing uncomplicated *P. falciparum* malaria in endemic countries Cochrane Database Syst Rev 2011 6 CD008122 doi:10.1002/1465 1858. CD008122. pub2 21735422 10.1002/14651858.CD008122.pub2 PMC6532563

[B11] Luchavez J Baker J Alcantara S Belizario V Jr Cheng Q McCarthy JS Bell D Laboratory demonstration of a prozone-like effect in HRP2-detecting malaria rapid diagnostic tests: implications for clinical management Malar J 2011 10 286 21957869 10.1186/1475-2875-10-286 PMC3214175

[B12] Gillet P Scheirlinck A Stokx J De Weggheleire A Chauque HS Canhanga OD Tadeu BT Mosse CD Tiago A Mabunda S Bruggeman C Bottieau E Jacobs J Prozone in malaria rapid diagnostics tests: how many cases are missed? Malar J 2011 10 166 21676264 10.1186/1475-2875-10-166 PMC3141590

[B13] Baker J Ho MF Pelecanos A Gatton M Chen N Abdullah S Albertini A Ariey F Barnwell J Bell D Cunningham J Djalle D Echeverry DF Gamboa D Hii J Kyaw MP Luchavez J Membi C Menard D Murillo C Nhem S Ogutu B Onyor P Oyibo W Wang SQ McCarthy J Cheng Q Global sequence variation in the histidine-rich proteins 2 and 3 of *Plasmodium falciparum*: implications for the performance of malaria rapid diagnostic tests Malar J 2010 9 129 20470441 10.1186/1475-2875-9-129 PMC2893195

[B14] Kemp DJ Thompson JK Walliker D Corcoran LM Molecular karyotype of *Plasmodium falciparum*: conserved linkage groups and expendable histidine-rich protein genes Proc Natl Acad Sci U S A 1987 84 7672 7676 3313401 10.1073/pnas.84.21.7672 PMC299362

[B15] Walker JA Dolan SA Gwadz RW Panton LJ Wellems TE An RFLP map of the *Plasmodium falciparum* genome, recombination rates and favored linkage groups in a genetic cross Mol Biochem Parasitol 1992 51 313 320 1349423 10.1016/0166-6851(92)90081-t

[B16] Pologe LG Ravetch JV Large deletions result from breakage and healing of *P. falciparum* chromosomes Cell 1988 55 869 874 3056622 10.1016/0092-8674(88)90142-0

[B17] Scherf A Mattei D Cloning and characterization of chromosome breakpoints of *Plasmodium falciparum*: breakage and new telomere formation occurs frequently and randomly in subtelomeric genes Nucleic Acids Res 1992 20 1491 1496 1579440 10.1093/nar/20.7.1491 PMC312228

[B18] Wellems TE Walliker D Smith CL do RV Maloy WL Howard RJ Carter R McCutchan TF A histidine-rich protein gene marks a linkage group favored strongly in a genetic cross of *Plasmodium falciparum* Cell 1987 49 633 642 2884039 10.1016/0092-8674(87)90539-3

[B19] Gamboa D Ho MF Bendezu J Torres K Chiodini PL Barnwell JW Incardona S Perkins M Bell D McCarthy J Cheng Q A large proportion of *P. falciparum* isolates in the Amazon region of Peru lack pfhrp2 and pfhrp3: implications for malaria rapid diagnostic tests PLoS One 2010 5 e8091 20111602 10.1371/journal.pone.0008091 PMC2810332

[B20] Dharia NV Plouffe D Bopp SE Gonzalez-Paez GE Lucas C Salas C Soberon V Bursulaya B Kochel TJ Bacon DJ Winzeler EA Genome scanning of Amazonian *Plasmodium falciparum* shows subtelomeric instability and clindamycin-resistant parasites Genome Res 2010 20 1534 1544 20829224 10.1101/gr.105163.110 PMC2963817

[B21] Akinyi S Hayden T Gamboa D Torres K Bendezu J Abdallah JF Griffing SM Quezada WM Arrospide N De Oliveira AM Lucas C Magill AJ Bacon DJ Barnwell JW Udhayakumar V Multiple genetic origins of histidine-rich protein 2 gene deletion in *Plasmodium falciparum* parasites from Peru Sci Rep 2013 3 2797 24077522 10.1038/srep02797 PMC3786299

[B22] Baker J McCarthy J Gatton M Kyle DE Belizario V Luchavez J Bell D Cheng Q Genetic diversity of *Plasmodium falciparum* histidine-rich protein 2 (PfHRP2) and its effect on the performance of PfHRP2-based rapid diagnostic tests J Infect Dis 2005 192 870 877 16088837 10.1086/432010

[B23] Maltha J Gamboa D Bendezu J Sanchez L Cnops L Gillet P Jacobs J Rapid diagnostic tests for malaria diagnosis in the Peruvian Amazon: impact of *pfhrp2* gene deletions and cross-reactions PLoS One 2012 7 e43094 22952633 10.1371/journal.pone.0043094 PMC3429466

[B24] Houze S Hubert V Le Pessec G Le Bras J Clain J Combined deletions of *pfhrp2* and *pfhrp3* genes result in *Plasmodium falciparum* malaria false-negative rapid diagnostic test J Clin Microbiol 2011 49 2694 2696 21543573 10.1128/JCM.00281-11 PMC3147824

[B25] Trouvay M Palazon G Berger F Volney B Blanchet D Faway E Donato D Legrand E Carme B Musset L High performance of histidine-rich protein 2 based rapid diagnostic tests in French Guiana are explained by the absence of pfhrp2 gene deletion in *P. falciparum.* PLoS One 2013 8 e74269 24086328 10.1371/journal.pone.0074269 PMC3781074

[B26] Koita OA Doumbo OK Ouattara A Tall LK Konare A Diakite M Diallo M Sagara I Masinde GL Doumbo SN Dolo A Tounkara A Traore I Krogstad DJ False-negative rapid diagnostic tests for malaria and deletion of the histidine-rich repeat region of the hrp2 gene Am J Trop Med Hyg 2012 86 194 198 22302847 10.4269/ajtmh.2012.10-0665 PMC3269266

[B27] Ramutton T Hendriksen IC Mwanga-Amumpaire J Mtove G Olaosebikan R Tshefu AK Onyamboko MA Karema C Maitland K Gomes E Gesase S Reyburn H Silamut K Chotivanich K Promnares K Fanello CI von Seidlein L Day NP White NJ Dondorp AM Imwong M Woodrow CJ Sequence variation does not confound the measurement of plasma PfHRP2 concentration in African children presenting with severe malaria Malar J 2012 11 276 22898068 10.1186/1475-2875-11-276 PMC3480887

[B28] Wurtz N Fall B Bui K Pascual A Fall M Camara C Diatta B Fall KB Mbaye PS Dieme Y Bercion R Wade B Briolant S Pradines B *Pfhrp2* and *pfhrp3* polymorphisms in *Plasmodium falciparum* isolates from Dakar, Senegal: impact on rapid malaria diagnostic tests Malar J 2013 12 34 23347727 10.1186/1475-2875-12-34 PMC3571878

[B29] Kumar N Pande V Bhatt RM Shah NK Mishra N Srivastava B Valecha N Anvikar AR Genetic deletion of HRP2 and HRP3 in Indian *Plasmodium falciparum* population and false negative malaria rapid diagnostic test Acta Trop 2013 125 119 121 23041541 10.1016/j.actatropica.2012.09.015

[B30] Padley DJ Heath AB Sutherland C Chiodini PL Baylis SA Establishment of the 1st World Health Organization International Standard for *Plasmodium falciparum* DNA for nucleic acid amplification technique (NAT)-based assays Malar J 2008 7 139 18652656 10.1186/1475-2875-7-139 PMC2518157

[B31] Yeboah-Antwi K Pilingana P Macleod WB Semrau K Siazeele K Kalesha P Hamainza B Seidenberg P Mazimba A Sabin L Kamholz K Thea DM Hamer DH Community case management of fever due to malaria and pneumonia in children under five in Zambia: a cluster randomized controlled trial PLoS Med 2010 7 e1000340 20877714 10.1371/journal.pmed.1000340 PMC2943441

[B32] Faucher JF Makoutode P Abiou G Beheton T Houze P Ouendo E Houze S Deloron P Cot M Can treatment of malaria be restricted to parasitologically confirmed malaria? A school-based study in Benin in children with and without fever Malar J 2010 9 104 20406492 10.1186/1475-2875-9-104 PMC2868860

[B33] d’Acremont V Malila A Swai N Tillya R Kahama-Maro J Lengeler C Genton B Withholding antimalarials in febrile children who have a negative result for a rapid diagnostic test Clin Infect Dis 2010 51 506 511 20642354 10.1086/655688

[B34] Mtove G Hendriksen IC Amos B Mrema H Mandia V Manjurano A Muro F Sykes A Hildenwall H Whitty CJ Reyburn H Treatment guided by rapid diagnostic tests for malaria in Tanzanian children: safety and alternative bacterial diagnoses Malar J 2011 10 290 21978238 10.1186/1475-2875-10-290 PMC3210100

[B35] Senn N Rarau P Manong D Salib M Siba P Robinson LJ Reeder J Rogerson S Mueller I Genton B Rapid diagnostic test-based management of malaria: an effectiveness study in Papua New Guinean infants with *Plasmodium falciparum* and *Plasmodium vivax* malaria Clin Infect Dis 2012 54 644 651 22198787 10.1093/cid/cir901

